# Cognitive rehabiliation for Parkinson's disease demantia: a study protocol for a pilot randomised controlled trial

**DOI:** 10.1186/s13063-016-1253-0

**Published:** 2016-03-22

**Authors:** John V Hindle, Tamlyn J Watermeyer, Julie Roberts, Anthony Martyr, Huw Lloyd-Williams, Andrew Brand, Petra Gutting, Zoe Hoare, Rhiannon Tudor Edwards, Linda Clare

**Affiliations:** Department of Care for the Elderly, Betsi Cadwaladr University Health Board, Llandudno, UK; North Wales Organisation for Randomised Trials in Health (NWORTH), Bangor University, Brigantia Building, Penrallt Road, Bangor, LL57 2AS UK; School of Psychology, Bangor University, Bangor, UK; Division of Mental Health and Learning Disabilities, Betsi Cadwaladr University Health Board, North Wales, UK; Centre for Research in Ageing and Cognitive Health (REACH), School of Psychology, University of Exeter, Exeter, UK; Centre for Health Economics and Medicines Evaluation (CHEME), Bangor University, Bangor, UK; Cefni Hospital, Llangefni, Anglesey UK

**Keywords:** Cognitive intervention, Cost-effectiveness, Quality of life, Parkinsonism

## Abstract

**Background:**

There is growing interest in developing non-pharmacological treatments to address the cognitive deficits apparent in Parkinson’s disease dementia and dementia with Lewy bodies. Cognitive rehabilitation is a goal-oriented behavioural intervention which focuses on improving everyday functioning through management of cognitive difficulties; it has been shown to be effective in Alzheimer’s disease. To date, no studies have assessed its potential efficacy for addressing the impact of cognitive impairment in people with Parkinson’s disease or dementia with Lewy bodies.

**Methods/design:**

Participants (*n* = 45) will be recruited from movement disorders, care for the elderly and memory clinics. Inclusion criteria include: a diagnosis of Parkinson’s disease, Parkinson’s disease dementia or dementia with Lewy bodies according to consensus criteria and an Addenbrooke’s Cognitive Examination – III score of ≤ 82. Exclusion criteria include: a diagnosis of any other significant neurological condition; major psychiatric disorder, including depression, which is not related to the patient’s Parkinson’s disease and unstable medication use for their physical or cognitive symptoms. A single-blind pilot randomised controlled trial, with concurrent economic evaluation, will compare the relative efficacy of cognitive rehabilitation with that of two control conditions. Following a goal-setting interview, the participants will be randomised to one of the three study arms: cognitive rehabilitation (eight weekly sessions), relaxation therapy (eight weekly sessions) or treatment as usual. Randomisation and treatment group allocation will be carried out by a clinical trials unit using a dynamic adaptive sequential randomisation algorithm. The primary outcomes are patients’ perceived goal attainment at a 2-months post-intervention assessment and a 6-months follow-up. Secondary outcomes include patients’ objective cognitive performance (on tests of memory and executive function) and satisfaction with goal attainment, carers’ perception of patients’ goal attainment and patients’ and carers’ health status and psychosocial well-being, measured at the same time points. Cost-effectiveness will be examined to explore the design of a larger cost-effectiveness analysis alongside a full trial.

**Discussion:**

This pilot study will evaluate the application of cognitive rehabilitation for the management of cognitive difficulties associated with Parkinson’s disease dementia and dementia with Lewy bodies. The results of the study will inform the design of a fully powered randomised controlled trial.

**Trial registration:**

ISRCTN16584442 DOI 10.1186/ISRCTN16584442 13 April 2015

## Background

Parkinson’s disease (PD) is a progressive neurodegenerative disorder which affects around 160 per 100,000 people in the UK. PD presents with motor symptoms of slowness, stiffness and tremor but is also associated with a spectrum of non-motor symptoms. These symptoms may be autonomic (swallowing, dribbling, constipation, sweating, urinary problems, dizziness) or neuropsychiatric (cognitive and behavioural impairment) in nature [1]. Neuropsychiatric symptoms predominate as PD progresses, with at least 80 % of people living with PD for more than 20 years fulfilling a dementia diagnosis [2]. Related to PD dementia is dementia with Lewy bodies (DLB), a condition whereby cognitive impairment precedes or occurs simultaneously with the development of the motor symptoms of PD [3]. A recent systematic review of 19 population-based and 10 clinic-based studies reported the mean prevalence of DLB to be 4.2 % and 7.5 % of all dementia cases in community (*n* = 2,178) and secondary care (*n* = 3,144) samples, respectively [4]. The neuropsychological profiles of PD dementia and DLB are similar and show subcortical or cortical patterns of impairment, with marked deficits in executive function, attention, visuospatial and memory abilities [5]. Patients’ cognitive impairments may have a significant impact on their own health, social care and quality of life as well as that of their carers [6, 7]. Care services are tasked with supporting the functional independence of these patients through alleviating or managing their neuropsychiatric and motor symptoms. This co-ordination may require input from both psychiatric and movement disorder specialists to address the complex needs of these conditions.

There is increasing interest in the effects of non-pharmacological interventions on cognitive functioning in neurological and psychiatric conditions. The efficacy of non-pharmacological therapies in Alzheimer’s disease (AD) and other dementias has been systematically studied, but it is not clear what the evidence is for efficacy in PD. In a systematic review of efficacy of non-pharmacological therapies for AD, recommendations were made for cognition-focused and multi-component interventions [8]. While cognitive training, the repeated practice of specific cognitive tasks, has not been found effective for people with mild dementia, cognitive rehabilitation (CR) has shown preliminary promise [9]. CR is a more individualised approach in which strategies to address personally relevant goals are devised and implemented [10]. The aim of rehabilitation is to enable people who are experiencing disability resulting from illness or injury to function at their optimum level, and ‘cognitive rehabilitation’ refers to the rehabilitation of people who have cognitive impairments. Therefore, CR aims to help people with early-stage dementia make the most of their memory and cognitive functioning despite the difficulties they are experiencing [11]. CR involves identifying and addressing individual needs and goals, which may require strategies for taking in new information or methods of compensating such as using memory aids. Activities may be targeted at improving individual cognitive deficits, compensating for the deficits, or developing adaptive methods to promote independence in instrumental activities of daily living. CR has been shown to be effective in AD in a single-blind randomised controlled trial comparing CR with relaxation therapy (RT) and treatment as usual (TAU) [12]. All participants were able to identify goals and rate their own performance and satisfaction with performance. CR produced significant improvements in goal performance and satisfaction, whereas there was no change in the other groups. The trial ’Goal-oriented cognitive rehabilitation in early-stage dementia: multi-centre single-blind randomised controlled trial (GREAT)’ has been funded by the HTA (HTA reference 11/15/04) and is currently underway [13]. This trial (ISRCTN21027481) aims to obtain definitive evidence about whether goal-oriented CR is a clinically effective and cost-effective intervention for people with early-stage AD, vascular or mixed dementia and their carers, but it does not include PD dementia or DLB.

Pharmacological treatment for the cognitive-behavioural symptoms associated with PD dementia and DLB includes the use of acetylcholinesterase inhibitors. However, due to their possible side effects (such as tremor, syncope or bradycardia) [14], these drugs may be unsuitable for many patients. There is therefore a need to consider non-pharmacological treatments which may help support the management of dementia in PD and DLB. In a recently completed systematic review of non-pharmacological therapy for enhancing cognition in PD, there were no studies assessing the effects of non-pharmacological interventions in PD dementia or DLB [15]. Similarly, published economic evaluations have focused on modelling the cost-effectiveness of pharmacological treatments for PD [16]. The choice of interventions needs to reflect what people with dementia and family members say troubles them and consider the help that would be beneficial in daily life. CR is a therapy which potentially addresses these issues by empowering people with dementia and family members to identify the goals of their own treatment.

This is the first trial of a cognition-focused intervention in PD dementia and DLB and the first study of CR in PD dementia and DLB. The feasibility of this type of cognitive intervention in AD is already established [12]. Therefore, this pilot study seeks to gain an indication of possible effect sizes for the primary outcomes as they are applied in the context of PD and DLB. It also aims to assess the appropriateness of the current secondary outcome measures, and establish other parameters for a future full trial, such as the appropriate method of measuring costs and effects for a cost-effectiveness analysis. CR will be compared with treatment as usual and with an active control condition involving relaxation therapy, providing the same amount of therapist time and attention. The hypothesis is that goal performance will improve significantly in the CR group compared with the relaxation therapy or treatment as usual groups. The other objectives of the pilot study are as follows:

(i) To assess the usefulness of outcome measures, particularly the ratings of goal attainment, and provide information on effect sizes to inform power calculations for a definitive multi-centre randomised controlled trial of CR in PD dementia and DLB.
(ii) To explore the usefulness of routine involvement of therapists in the management of cognitive problems in these conditions to improve access to psychological therapies.
(iii) To gain a preliminary indication of the transfer of treatment effects into everyday life for the patient.
(iv) To conduct a pilot assessment of cost-effectiveness which will support the development of a larger trial providing definitive evidence on cost-effectiveness.

## Methods/design

### Study settings

Participants will be recruited from movement disorders and memory clinics within the Betsi Cadwaladr University Health Board (BCUHB), Wales, UK. Data collection will take place in the participants’ homes to accommodate possible fatigue and physical disability as well as to reflect the context in which cognitive difficulties will be experienced and managed in their everyday lives.

### Ethics and governance

Ethical approval for the study has been obtained from the Wales Research Ethics Committee 5 (13/WA/0340). Informed written consent will be obtained from all participants (patients and carers) prior to entry into the trial. As potential participants will be in the early stages of dementia, they are expected to have the capacity to consent to participation, and loss of capacity to consent during the course of participation is expected to be very infrequent. On entry to the trial, participants will be asked whether, should they lose capacity to consent, they are willing to continue to be included in the trial and to have their data used.

### Sample size

**Fig. 1:**
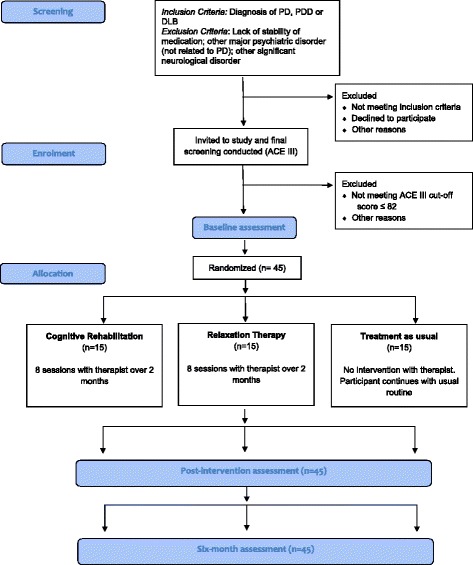
CORD PD Study CONSORT-style flowchart. Recruitment began in April 2015 and will continue until May 2016. The study end date is January 2017

As this is a pilot study, it is not intended to be fully powered for detection of statistically significant effects, but is intended to provide information that will inform the sample size calculation for a full randomised trial. Evidence from a pilot trial using the same design for participants with AD [12] indicates that 24 people in each group gave a standardised effect size > 1 post intervention with 7 % attrition. Through collaboration with the Clinical Trials Unit, North Wales Organisation for Randomised Trials in Health (NWORTH), it was agreed that 15 in each condition will provide enough information about the variability of the outcome and likely attrition rate within the population to inform the sample size calculation. The target sample size will therefore be 45 in total recruited from across the target population (Fig. 1). Studies show a prevalence of dementia in people with PD of 30 %. There are approximately 1,500 people with PD attending the movement disorder clinics in BCUHB, giving a likely prevalence of 450 people with dementia with approximately 150–200 fulfilling the inclusion criteria. PD dementia constitutes 5 % of all people with dementia, whereas DLB is more common, constituting up to 20 % of referrals for dementia in specialist centres [17]. DLB is less common in general memory clinics in BCUHB and may be under-diagnosed (personal communication from Professor Bob Woods) but is more often seen in the movement disorder clinics. Taking these factors into account, the number of eligible people with DLB in BCUHB is likely to be 100, bringing the total eligible population to 250–300.

### Randomisation

Randomisation will be completed by NWORTH using a dynamic adaptive sequential randomisation algorithm [18]. Allocation will be on an equal ratio between the three groups, stratifying for diagnosis (DLB/PD), gender and age (≤69, 70+). Researchers collecting follow-up data will be blind to the randomisation outcome.

### Participants

#### Inclusion criteria

Participants will have either a diagnosis of PD according to the UK PD Brain Bank Diagnostic Criteria [19] and a diagnosis of PD dementia according to the Movement Disorder Society consensus criteria [20, 21] or a diagnosis of DLB according to the consensus criteria [22]. Participants will have mild to moderate dementia, indicated by an Addenbrooke’s Cognitive Examination – III (ACE-III) [23] score ≤82. Participants will ideally have a carer or family member who is willing to participate, but this is not an absolute requirement. Where participants are using medication for their parkinsonism and/or cognitive symptoms (including acetylcholinesterase inhibitors), this use should be stable for four weeks prior to commencement with no changes planned for the duration of the trial. Any unplanned changes in medication during the trial will be documented.

#### Exclusion criteria

Exclusion criteria are lack of stability of PD medications, cognitive enhancers or psychotropic medication, substantial additions to medication in the four weeks before the trial or planned changes during the period of the trial, other major psychiatric disorder not related to PD, major depression and other significant neurological disease.

#### Goal setting

All participants will collaboratively agree on problem areas or areas that they would like to manage better, formulated in the form of up to three personal rehabilitation goals using the Bangor Goal-Setting Interview (BGSI) [24]. They will all rate their own performance on these goals at initial and follow-up assessments, but only the CR group participants will work with a therapist to address these goals.

### Interventions

#### CR with the therapist

Goals identified at the initial assessment will be communicated to the therapist, who will use them as the starting point for therapy and further operationalise and refine them if necessary. Eight individual one-hour sessions of CR will be delivered at home over 2 months. Carers will be involved in part of each session where possible. Goals will be introduced one at a time, in a flexible manner depending on rate of progress. The participant, with the carer (where available), will work on the selected goals between sessions following an agreed schedule of activities. The therapist will use a variety of evidence-based strategies and supporting components dependent on the nature of the goals set (see Table 1 for examples of possible goals and strategies). Strategies for managing practical situations and cognitive difficulties as well as anxiety symptoms will be introduced across the sessions. These sessions will build on participants’ existing abilities and emphasise a problem-solving approach to goal attainment, where steps to success are specified and possible solutions are tested. Compensatory strategies may be used (for example, calendars, diaries, reminders) as well as restorative strategies for retaining new information or improving recall (such as mnemonics or spaced retrieval). Progress with each goal will be reviewed and the strategies adopted will be adjusted as necessary on a weekly basis. In-session ratings of goal performance will be made by participants, carers and the therapist to evaluate progress. The therapist is a qualified occupational therapist working within NHS Wales and is experienced in providing neurorehabilitation interventions for adult patient populations. Trial-specific training is provided on an ongoing basis, and the therapist’s adherence to the treatment protocols will be monitored through therapy logs and regular supervision sessions.

**Table 1:** Examples of goals and strategies employed for cognitive rehabilitation

**Goal**	**Strategies employed**
“I will be able to use the iPad to send emails”	*Compensatory*: Use cue card for step-by-step instructions. Use of iPad stylus pen is easier than finger. Practice alongside carer or therapist.
	*Restorative*: Action-based learning: keep iPad and cue card visible to prompt use. Use flowchart to break activity into smaller steps.
“I will be able to find my purse, keys, hearing aid and reading glasses”	*Compensatory*: Identify places for items to be left. Place prompt cards in other likely places where participant leaves items to encourage appropriate placement of items. Place prompt card by front door to ensure participant has items before leaving house. Use colour to support object recognition: coloured cord put on glasses; coloured ribbon put on purse.
“I will be sociable during mealtimes and appear to take an interest in conversation rather than just concentrating on eating”	*Compensatory*: Hearing assessment and new hearing aid. Notebook to record information participant could use in conversation.
	*Restorative*: Increasing awareness of other peoples’ perception of the participant. Active listening alternately with eating and putting knife and fork down to make eye contact, listen and converse.

*Note*: Adapted into a table from Johns, R., Page, P., Pool, J., Besso, E., Evans, S., Green, J., Tranah, A., Clare, L. (2015, July). *Goal*-*oriented cognitive rehabilitation*: *improving the experience of dementia*. Poster presented at the 39th College of Occupational Therapists Annual Conference, Brighton, UK.

### Comparators

CR will be compared with relaxation therapy (RT) and treatment as usual (TAU). RT is included as an active control condition involving equivalent therapist time and attention, since PD and related conditions are known to be very susceptible to placebo effects by modulating dopamine release in relation to expectation [25]. RT will consist of eight individual sessions of one hour’s duration in participants’ own homes based on the eight- session protocol used in the AD pilot study [12]. After an initial ‘getting to know you’ session where the therapeutic model is explained, each session has a theme which includes music, pictures, radio and television, sensory stimulation and humour before a final consolidation. Each session includes consolidation of the last one, a mood checklist and some homework. TAU will consist of usual medication and any other services apart from specific programmes of CR or other cognition-focused intervention. TAU may include routine monitoring by the movement disorder clinic or memory clinic, information provision, attendance at drop-in groups or support groups, or carer participation in support groups. Participants in the treatment conditions will also have access to any such resources that they might use over the course of the trial; the use of such resources will be noted by the researcher at the study visits.

### Outcomes

#### Primary outcomes

Participants’ performance on goals identified through the BGSI is rated with the researcher at the beginning and at the follow-up visits. These ratings of goal performance are the primary between-group comparators. The BGSI [24] is a structured interview in which respondents are asked to identify areas of their daily activities that are difficult to do to their own satisfaction, and in which they would like to see improvements. For each goal, performance is rated on a 1 – 10 scale (1 = unable to perform; 10 = fully able to perform) and mean levels of performance are calculated by summing the individual goal ratings and dividing by the number of goals. The participants’ ratings will be made at the initial assessment, at the end of the intervention period (or equivalent time for TAU group), and at 6 months post-randomisation.

#### Secondary outcomes

The assessment of secondary outcomes will assist in determining the most appropriate outcome measures to be used for a larger trial. For people with PD dementia, these cover the domains of cognition, mood, behaviour, everyday functional activity, motor severity, quality of life, self-efficacy and carer ratings (where available) of patients’ performance on goals identified during the BGSI. Within-group data from CR sessions on goal setting and performance will be assessed to give information on factors such as the types of goals that participants choose and which types of goals are addressed most effectively through the therapy. Also, for the CR group, patients, their carers and the therapist will make in-session ratings of goal attainment to provide concurrent evidence regarding changes in goal performance. Differences between these ratings of the patient’s goal attainment will also be examined across the rater groups. Carers’ outcomes include quality of life, stress and health.

Regarding cognition, global cognition will be measured using the ACE-III [23]. Assessments of executive function will be the Trail Making Test (TMT) and the letter fluency tests from the Delis-Kaplan Executive Function System (DKEFS) [26]. Attention will be assessed using the Test of Everyday Attention (TEA) [27]. Memory will be assessed using the Rivermead Behavioural Memory Test (RBMT) [28], Story Recall subtest.

Mood will be assessed using the Hospital Anxiety and Depression Scale (HADS), which contains subscales for anxiety and depression and is validated in PD [29].

Behavioural assessment will include delusions and hallucinations measured using the Neuropsychiatric Inventory Questionnaire (NPI-Q) [30]. The Unified Parkinson’s Disease Rating Scale (UPDRS) [31] will be used to assess the motor symptoms of parkinsonism in PD and DLB (part 111) and also to assess function and activities of daily living.

Quality of life will be assessed in PD and DLB using the abbreviated Parkinson’s Disease Questionnaire (PDQ-8) [32] and, in order to have a direct comparison, the World Health Organisation Quality of Life – BREF (WHOQOL-BREF) [33], which has been validated in PD [34, 35] will also be used with carers.

In order to assess a general sense of perceived self-efficacy, the potential to influence one’s situation through one’s own actions, the General Self-Efficacy Scale (GSE) [36] will be used.

The BGSI also requires carers to rate the patients’ level of performance on a 1 – 10 scale (1 = unable to perform; 10 = fully able to perform). Carer ratings will be recorded at the baseline, post-intervention and follow-up researcher visits.

For the CR group only, a simplified goal attainment scaling procedure [37] will be used to obtain in-session ratings of the patient’s goal attainment by the participant, the carer and the therapist. These will be obtained at the first, fourth and eighth (final) therapy sessions.

Carers’ stress will be assessed using the 15-item dementia-specific Relatives Stress Scale (RSS) [38].

Service receipt during the intervention period, including dementia-specific services, monitoring, and interventions provided by movement and memory clinics, will be documented for all participants. All participants will be free to access services such as those offered by Parkinson’s UK, and the extent of this will be recorded.

Cost-effectiveness will be piloted using measures of health status, the EQ-5D-3 L [39]. We will develop and use a Client Service Receipt Inventory (CSRI) [40] specific to this patient group that can be used later in a full trial to assess health care utilisation. Other information collected will include gender, age, the relationship between the person with PD dementia and their carer and whether they live together, age of onset of PD, PD dementia, or DLB, Hoehn and Yahr PD severity [41]. Medication, educational level, social class, and co-morbidities will be recorded to examine effects of demographic and social variables on treatment efficacy.

### Procedures

The baseline assessment will be conducted once participants have given informed consent. All participants will engage in a goal-setting interview at the baseline visit. Participants will then be randomised and results of the randomisation will be sent to the therapist. Participants allocated to CR or RT will receive eight weekly visits from the therapist over a 2-month period. The post-intervention assessment for all participants will be conducted 2 months after randomisation. The final follow-up assessment will take place 6 months after randomisation.

The researcher taking the measures will be blind to allocation, as will the data analysts. Blinding is achieved as follows: following the completion of the baseline visits, the researcher triggers randomisation through a secure web-based system hosted by NWORTH. This system randomisation is performed independently of the data analysis team procedures. The outcome of the randomisation event is emailed to the therapist only. The therapist contacts the participants, informing them as to which treatment arm they have been allocated. The importance of maintaining blinding will be emphasised in the training for both researcher and therapist. Since participants will be aware of their treatment allocation, they will be asked not to reveal to the researcher whether or not they were visited by the therapist. Following each follow-up assessment, the researcher will record to which condition she believes the participant has been allocated and will rate her level of certainty regarding this allocation. If participants overtly reveal their allocation to the researcher, this will be noted. Sensitivity analyses will determine whether these ratings or knowledge influenced the scoring of participant data. Analyses will be adjusted to counterbalance any evident bias.

### Data management and analysis

Quantitative research data will be collected and recorded onto paper-based questionnaire booklets and will be entered via a web interface to the MACRO participant measures database held at NWORTH. Initial data management and cleaning will be conducted by the research team in the study centre, and the secondary cleaning, preparation and extraction of the datasets for analysis will be conducted by NWORTH statisticians. Analysis will be completed for each outcome measure at post-intervention (2 months) and at the 6-months follow-up using an analysis of covariance model, with baseline scores as a covariate, allocated group as the condition factor and the stratification variables as fixed factors. Confidence intervals will be calculated for the standardised mean difference.

### Cost-effectiveness analysis

Based on the Medical Research Council guide to the evaluation of complex interventions [42], we will conduct a pilot cost-effectiveness analysis to explore how best to collect information on intervention costs and service use by this specific group of participants and relate this information to primary and secondary outcome measures. We will take a broad public sector, multi-agency perspective, mapping out relevant stakeholders and the full potential range of possible costs and consequences to be measured. This will identify potential areas for cost-savings in the long-term management of people with Parkinson’s disease dementia and dementia with Lewy bodies. We will examine the feasibility of using the EQ-5D-3 L (researcher administered) to provide a pilot cost per quality-adjusted life year (QALY) calculation. We will make best use of routine service utilisation, cost data and national unit cost data. The researcher-administered CSRI, specially tailored using the Database of Instruments for Resource Use Measurement (DIRUM) database of CSRIs [43], will gain further information about the type and frequency of service use. We will examine whether a cost consequence or cost utility analysis would be most appropriate for extension to a full trial.

## Discussion

This study will provide preliminary indications of efficacy for CR in the management of dementias associated with Parkinson’s disease. In addition, the study will provide information on the appropriateness of the current outcome measures and will pilot cost-effectiveness measures, producing estimates of relative cost-effectiveness. The results of the study will support the selection of outcome measures and the estimation of parameters for a larger RCT of CR in PD dementia.

There are no known risks associated with CR. It is possible that some participants may find it challenging to confront their difficulties, but the therapist will provide support as they engage in this process, and the intervention protocol incorporates attention to managing emotional reactions. As this is a short pilot trial and there have been no other trials of CR in PD or DLB, it is not considered unethical to withhold CR from the comparator groups. The research team will be trained to be alert to any concerns about participants’ well-being. If there are serious concerns about a participant, they will be referred, wherever possible with the participant’s permission, to the clinician responsible for the participant’s care. Blinding of the researcher, who assesses participants’ outcomes, will be maintained through strict procedures. Any deviation from these procedures will be recorded, and blinding success will be examined at the end of the study. As this is a single-blind study, and participants are aware of their treatment allocation, this may introduce bias through demand characteristics. An active control condition (RT) will help assess if any improvements found for the experimental group (CR) resulted from placebo effects. A key component of this pilot trial is to establish whether CR shows indications of effectiveness for PD dementia or DLB [13]. As CR will be conducted by a therapist in a naturalistic setting (in the patients’ own homes), the pilot will assess the practicalities of its intended delivery and direct strategies for its implementation in future studies or care practice.

The results of this study will contribute to the development of non-pharmacological care approaches to cognitive impairment in PD, promoting patients’ functional independence, improving their well-being and that of their carers. The majority of movement disorder clinics in the UK do not have immediate access to psychological services; the results of this work may potentially foster future co-operation of multidisciplinary teams for PD services in NHS Wales and beyond.

### Trial status

The CORD-PD trial started on 1 January 2015. Recruitment commenced in April 2015 and will continue until May 2016. The end date for the trial is January 2017.

## Abbreviations

ACE-III: Addenbrooke’s Cognitive Examination – III
ACE-R: the revised ACE
AD: Alzheimer’s disease
BCUHB: Betsi Cadwaladr University Health Board
BGSI: Bangor Goal-Setting Interview
CDR: Clinical Dementia Rating
COPM: Canadian Occupational Performance Measure
CR: cognitive rehabilitation
CSRI: Client Service Receipt Inventory
DIRUM: Database of Instruments for Resource Use Measurement
DKEFS: Delis-Kaplan Executive Function System
DLB: dementia with Lewy bodies
GREAT: Goal-oriented cognitive rehabilitation in early-stage dementia: multi-centre single-blind randomised controlled trial
GSE: General Self-Efficacy Scale
HADS: Hospital Anxiety and Depression Scale
NPI-Q: Neuropsychiatric Inventory Questionnaire
NWORTH: North Wales Organisation for Randomised Trials in Health
PD: Parkinson’s disease
PDQ-8: the abbreviated Parkinson’s Disease Questionnaire
QALY: Quality-adjusted life year
RBMT: Rivermead Behavioural Memory Test
RSS: Relatives Stress Scale
RT: relaxation therapy
TAU: treatment as usual
TEA: Test of Everyday Attention
TMT: Trail Making Test
UPDRS: Unified Parkinson’s Disease Rating Scale
WHOQOL-BREF: World Health Organisation Quality of Life – BREF
