# Erratum to: Cognitive rehabiliation for Parkinson’s disease dementia: a study protocol for a pilot randomised controlled trial

**DOI:** 10.1186/s13063-017-1849-z

**Published:** 2017-03-23

**Authors:** John V. Hindle, Tamlyn J. Watermeyer, Julie Roberts, Anthony Martyr, Huw Lloyd-Williams, Andrew Brand, Petra Gutting, Zoe Hoare, Rhiannon Tudor Edwards, Linda Clare

**Affiliations:** 1 Department of Care for the Elderly, Betsi Cadwaladr University Health Board, Llandudno, UK; 2 North Wales Organisation for Randomised Trials in Health (NWORTH), Bangor University, Brigantia Building, Penrallt Road, Bangor, LL57 2AS UK; 3 School of Psychology, Bangor University, Bangor, UK; 4 Division of Mental Health and Learning Disabilities, Betsi Cadwaladr University Health Board, North Wales, UK; 5 Centre for Research in Ageing and Cognitive Health (REACH), School of Psychology, University of Exeter, Exeter, UK; 6 Centre for Health Economics and Medicines Evaluation (CHEME), Bangor University, Bangor, UK; 7 Cefni Hospital, Llangefni, Anglesey UK

## Erratum

The title contains an error in the original publication [1]. The correct version: “Cognitive rehabilitation for Parkinson’s disease dementia: a study protocol for a pilot randomised controlled trial”.
